# Use of historic metabolic biotransformation data as a means of anticipating metabolic sites using MetaPrint2D and Bioclipse

**DOI:** 10.1186/1471-2105-11-362

**Published:** 2010-07-01

**Authors:** Lars Carlsson, Ola Spjuth, Samuel Adams, Robert C Glen, Scott Boyer

**Affiliations:** 1 Safety Assessment, AstraZeneca Research & Development, 431 83 Mölndal, Sweden; 2 Department of Pharmaceutical Biosciences, Uppsala University, 751 24 Uppsala, Sweden; 3 Unilever Centre for Molecular Sciences Informatics, University Chemical Laboratory, Cambridge, CB2 1EW, UK

## Abstract

**Background:**

Predicting metabolic sites is important in the drug discovery process to aid in rapid compound optimisation. No interactive tool exists and most of the useful tools are quite expensive.

**Results:**

Here a fast and reliable method to analyse ligands and visualise potential metabolic sites is presented which is based on annotated metabolic data, described by circular fingerprints. The method is available via the graphical workbench Bioclipse, which is equipped with advanced features in cheminformatics.

**Conclusions:**

Due to the speed of predictions (less than 50 ms per molecule), scientists can get real time decision support when editing chemical structures. Bioclipse is a rich client, which means that all calculations are performed on the local computer and do not require network connection. Bioclipse and MetaPrint2D are free for all users, released under open source licenses, and available from http://www.bioclipse.net.

## Background

Metabolic stability is a critical aspect in the drug discovery process. A candidate drug faces a system that has evolved to prevent exposure of foreign compounds in the body by metabolising enzymes. Xenobiotic metabolism mainly occurs in two places; the gut epithelia and the liver. The mechanisms are quite well understood, but there is still a tremendous variation in reactions that lead to biotransformations. Many enzymes can carry out the same reactions on a variety of substrates. All together the metabolising systems are hard to model with high accuracy.

The currently available predictive tools are usually tuned and validated against literature data, and few available methods rapidly present the results in a simple interface. A simple database search only shows the exact reported literature results and quite cumbersome queries are needed to retain any information at all about probable sites of metabolism [1]. Much of the data in the literature have been collected by Symyx(R) http://www.symyx.com and they provide a tool, ISIS, to search for structures and other entities to retrieve putative metabolic sites. The data have been curated and is regarded to be of high quality. Another tool is Metasite [2] which predicts metabolic sites in a semi-deterministic fashion. Another method uses historic metabolic biotransformation data from the literature exclusively [3]. These methods are all fairly accurate [4] and provide chemists with results within a reasonable time. However, none of them is interactive and allows a user to get instant updates during editing of chemical structures, and some of them are only available under expensive licenses that restrict their availability. Also available is Meteor [5], which is easy to use and gives reasonably clear results. But, it generates many possible metabolites that perhaps are not all probable. To fully make use of all different features in some of the tools, a substantial amount of computer knowledge is required. There is a need for fast, reliable, and easy to use method for site-of-metabolism predictions.

This paper describes an interactive tool for prediction of metabolic sites that can run on a desktop and be managed by "wet-lab chemists". It has been built by combining two existing applications Bioclipse [6] and MetaPrint2D based on [3]. The next section explains how the two components work and how they have been combined, followed by a section where results are presented to illustrate its use and performance, ending with discussions about the implications.

## Methods

### MetaPrint2D

MetaPrint2D is a tool for predicting the sites of a molecule that are most likely to undergo Phase I metabolism, based on their similarity to known sites of metabolism and sites that are known not to be metabolised. The method builds on a database of atom environments found in molecules known to undergo metabolic transformation, such as the data found in the Symyx(R) (previously MDL) Metabolite database http://www.symyx.com/, which contains over 80.000 metabolic transformations of xenobiotics, curated from reports in scientific literature. MetaPrint2D contains a complete enumeration of the atom environments occurring in reactant molecules in the Metabolite database, together with counts of the number of occurrences of the atom environment at a reaction centre, and anywhere in the database, whether at a reaction centre or not.

In order to make predictions on a molecule the atom environments in the molecule are calculated, and the database is searched for similar environments. An occurrence ratio - measuring how often this or a similar environment has been found at a reaction centre, relative to how many times it has been observed in total -is then calculated for each atom in the molecule. These ratios represent the relative likelihood of metabolism occurring at each atom; no prediction is made as to whether the molecule does indeed undergo metabolism, rather a ranking of the likelihood of metabolism occurring at each site in the molecule is produced. In order to standardise the output, these ratios are then scaled so that the molecule's most likely site of metabolism gets a normalised occurrence ratio of one.

#### Preprocessing

The MetaPrint2D data is generated through processing of the transformations found in the Symyx(R) Metabolite database. For each transformation, the differences between the structure of the reactant and product are identified: groups added or eliminated, bonds broken or made and bonds whose order has changed. In order to simplify the results, only Phase I additions (defined as the addition of a single oxygen atom; covering hydroxylation, oxidation and epoxidation), and eliminations (for example dealkylation, ester and amide hydrolysis) are retained. For an addition, the atom neighbouring the added oxygen is marked as a reaction centre. In the case of an elimination, a bond gets broken, and both atoms connected by the bond are considered to be reaction centres.

**Figure 1:**
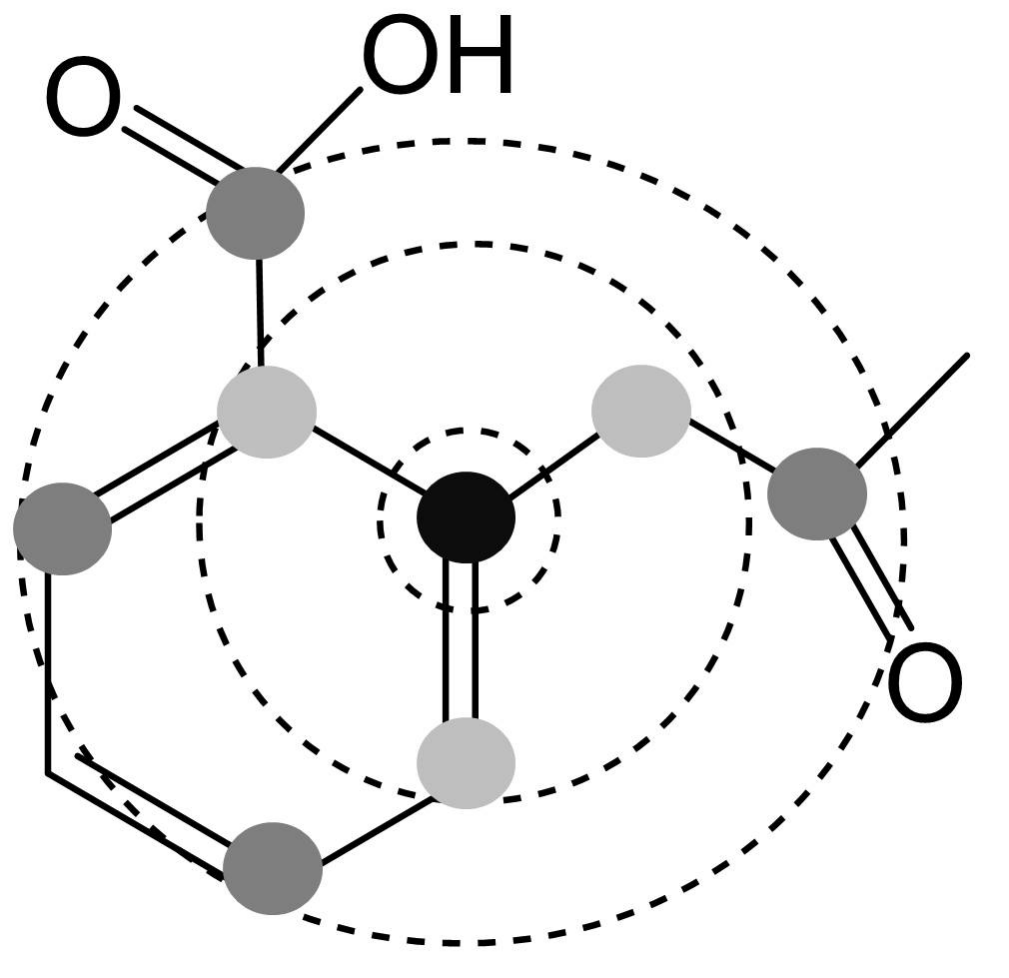
**Circular fingerprints**. An illustration of a three-level atom environment. The atom the environment is calculated for is at the centre of the environment, labelled black in this figure, and the atoms in the second and third layers are labelled light and dark grey, respectively.

The preprocessing is continued by a generation of circular fingerprints [7,8] for each reaction centre and also for every atom in the reactants. Circular fingerprints (also known as spherical fingerprints) represent the chemical environment of an atom in a form that can be processed by a computer (Figure 1). These fingerprints include the number of occurrences of atoms of various types in each of a series of concentric layers radiating out from a central atom; the first layer contains the atom the environment is centred on, and each subsequent layer contains the immediate neighbours of the atoms in the previous layer, so the second layer contains those atoms directly bonded to the central atom, and the third layer contains the atoms directly bonded to those atoms in the second layer. An atom environment can extend to an arbitrary number of layers. MetaPrint2D utilises environments up to six layers in depth. Rather than describing atoms by their chemical element, they were described using the implementation of Tripos forcefield atom types in the Chemistry Development Kit [9], which combine the atom's chemical element with its hybridisation and aromaticity. The used Sybyl atom types were C.3, C.2, C.ar, C.1, N.3, N.2, N.1, O.3, O.2, S.3, N.ar, P.3, H, Br, Cl, F, I, S.2, N.pl3, LP, Na, K, Ca, Li, Al, Du, Si, N.am, S.O, S.O2, N.4, O.CO2, and C.cat. Thus for each unique circular fingerprint the occurrence as a reaction centre as well as the overall occurrence is stored in the following sparse format (in our Java implementation):

int numberOfFingerprints   //All fingerprints from the

                           //transformation db.

byte numberOfLevels        //Fingerprint levels.

                           //We only use six.

 

//For each fingerprint

byte numberOfNonZeroEntries // All existing atoms.

 

//For each non-zero entry

byte index // The index representing the Sybyl atom type.

byte value // The occurrence of a specific Sybyl atom type

 

int rc // Number of occurrencs as reaction centre.

int oc //Number of occurrences overall.

byte errorCheckingByte // Always set to a unique value.

#### Predictions

The second stage of the proposed method is what the user experiences. It starts by reading a file of preprocessed transformation data, putting all fingerprints with their associated information, such as number of occurrences as reaction centres and number of occurrences overall in reactants, in memory. All atoms of a query molecule undergo the same procedure to generate circular fingerprints based on Sybyl atom types and to allow for comparison to the reactants of the underlying transformation database. In addition to the steps of generating the circular fingerprints, a hashcode, *h*, is generated for each fingerprint level by the following recurrence sequence:

where *s*_*i *_is the sum of a specific Sybyl atom type, represented by the index *i *and *α *is an integer constant. The preprocessed transformation data is searched by collecting all circular fingerprints with identical hashcodes, for a predefined number of levels, to each atom fingerprint in a query molecule. The hashcode is a compact representation of a fingerprint which is optimised for comparisons, where two identical hashcodes are not necessarily representing identical fingerprints but two identical fingerprints have identical hashcodes. If an atom fingerprint in a query molecule, *F*^*q*^, and a transformation molecule atom fingerprint, *F*^*t*^, are considered to be similar the following needs to be true:

where *N *is the number of fingerprint levels required for an exact match, *w*_*l *_is a weight that can be set for each fingerprint level and *ε *is a similarity threshold. By searching the transformation data fingerprints using the hashcodes, the number of fingerprints searched using the similarity operator in Equation 3 is substantially reduced without any loss of information. The sum of both the reaction centre occurrences and the overall occurrences for all similar transformation data fingerprints are stored for each query atom, respectively. These ratios are also normalised by the greatest ratio within a query molecule for an easy overview and a possible rank order for predicted sites of metabolism.

#### Bioclipse

Bioclipse [6] is a graphical workbench for life science which is equipped with tools required for many common cheminformatics tasks, such as loading and converting between file formats, editing of chemical structures, interactive visualisation in 2D/3 D, and editing of compound collections. Bioclipse is however not limited to this; its extensible architecture enables it to be extended into virtually any direction, and has been used in such various fields as drug discovery, bioinformatics, spectral analysis, high throughput screening, systems biology, and general chemistry education. As a rich client application, Bioclipse is downloaded and installed onto desktop computers, and is able to utilise their full power while also taking full advantage of new e-Science technologies, such as on-line (Web) services, high-performance computing, and networked databases. Equipped with an advanced provisioning system, it is very easy to publish updates for Bioclipse. This not only simplifies maintenance, but also enables users to stay up to date and always use the most current version of algorithms and data.

## Results and Discussion

Version 1.0 of of MetaPrint2D has been trained using the 2008.1 version of the Symyx(R) Metabolite database. Four different MetaPrint2D databases are currently provided, one generated from all the transformations in the Metabolite database, and others generated only using transformations found to occur in particular species - human, rat and dog. For the layered fingerprint hashcodes, α was set to 16 and N (number of exact levels) was set to 3.

A MetaPrint2D plugin for Bioclipse was created which adds several new options for visualising chemical structures. A specific toolbar and a menu allows the user to select one of the available MetaPrint2D databases (All, Human, Dog, or Rat), adds options for selecting search mode, and also provides means to execute the MetaPrint2D calculation and to visualise the result in the chemical structure. Currently the file formats MDL Molfile, Chemical Markup Language (CML), SMILES, and Structure Data File (SDF) are supported, and results can be visualised for single molecules or multiple structures in a table. It is also possible to execute MetaPrint2D in batch mode, and process for example SD files containing large collections of structures without visual inspection. MetaPrint2D is also accessible from the Bioclipse scripting language and can easily be integrated in custom Bioclipse scripts to automate tasks (for an example, see [10]).

**Figure 2:**
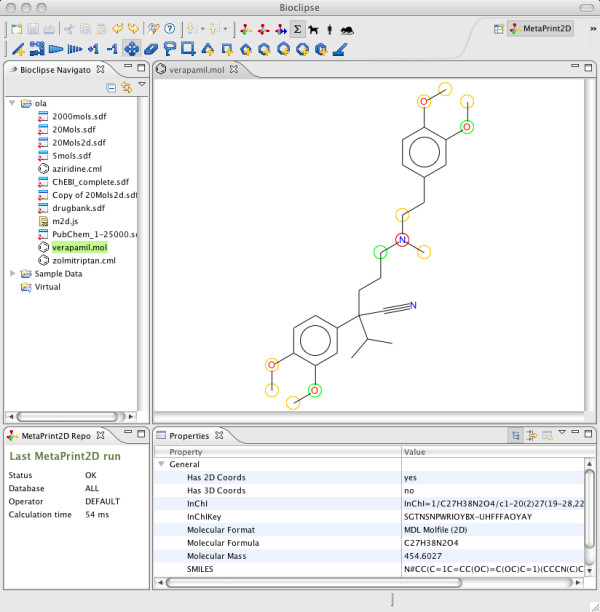
**MetaPrint2D in Bioclipse showing a prediction on the drug Verapamil**. Screenshot from Bioclipse with the results from a MetaPrint2D calculation on the drug Verapamil using the Standard search mode and the database ALL. Atoms coloured in red indicates a high likelihood of metabolism occurring at that site, yellow indicates medium, green low, and no colouring indicates very low or no likelihood of metabolism occurring at that site. Hovering over an atom reveals a tooltip showing the number of hits in the reaction centre set, the total number of hits, and the normalised ratio.

Running a MetaPrint2D prediction is easy within Bioclipse. The Navigator pane on the left hand side (Figure 2) allows for management of files and folders that can be operated upon in Bioclipse, and points to a place on the local hard disk. The Navigator is easily populated by drag and drop or cut and paste of files and/or folders, or using the Import functionality of Bioclipse. Opening a single molecule in the 2 D editor or multiple molecules in the Molecules Table of Bioclipse reveals the MetaPrint2D toolbar, allowing for MetaPrint2D calculations. The results are visualised so that atoms are coloured according to the likelihood of a metabolic site being centred on this atom: High: red, Medium: yellow, Low: green, and Very low is not coloured. In single molecule mode the molecule can be edited and by a click of a button the new prediction is computed and displayed, or automatic prediction is turned on and a new prediction is executed upon changing the structure. Figure 2 shows the drug Verapamil opened in Bioclipse, and coloured by MetaPrint2D analysis using the Standard search mode and the database ALL. Hovering over an atom reveals a tooltip showing the number of hits in the reaction centre set, the total number of hits, and the normalised occurrence ratio. It is simple to edit the chemical structure and update the visualisation; the process is near real-time which makes it very easy to explore changes to the structure that affect the likely site of metabolism.

**Figure 3:**
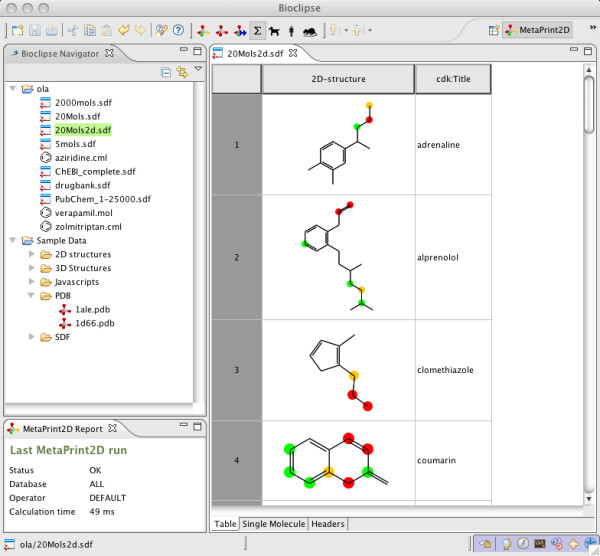
**MetaPrint2D in Bioclipse showing predictions on multiple molecules**. Screenshot from Bioclipse with the results from a MetaPrint2D calculation of an SD file illustrating four well known drugs using the Standard search mode and the database ALL. This gives an informative overview when working with collection of molecules, and it is simple to switch to editing individual molecules using the 2D-structure view.

It is straightforward in Bioclipse to invoke MetaPrint2D on files containing multiple molecules. Figure 3 shows an SD file opened and predicted in Bioclipse. The same colouring as in single molecule mode is produced, and the user can switch between this tabular view of several compounds to a more detailed view of a single compound by selecting the Single Molecule tab in the bottom of the table. Here the user can make desired structural changes to a compound, and then switch back to the tabular view and proceed to another compound in the collection. There is also the ability to execute MetaPrint2D in batch mode by right-clicking a file and selecting "Run MetaPrint2D". An SD file comprising 232 compounds (available in suppl. material) with 20 atoms on average was subjected to MetaPrint2D calculation in batch mode using Standard search mode and the database ALL. The file took 8.1s to process, which is an average of 34 ms per molecule. All results were measured on a MacBook Pro laptop using Bioclipse version 2.4, MetaPrint2D Bioclipse plugin version 1.0, MetaPrint2D version 1.0, and Symyx(R) Metabolite database 2008.1.

The source code for the MetaPrint2D algorithm is available from Sourceforge [11], and this version can be used as a standalone calculator called from the command line. The source code from the Bioclipse-MetaPrint2D plugin is available from Github [12], and a page describing the Bioclipse integration is also available [13]. A web page for demonstrating MetaPrint2D is available [14]; note however that the MetaPrint2D version on the web page might differ from the version included in the Bioclipse-MetaPrint2D plugin and what is described herein.

## Conclusions

We here present MetaPrint2D, which is an improved algorithm for site-of-metabolism prediction. The efficient use of layered, hashed circular fingerprints significantly improves the speed of predictions over Boyer et al. (2007), and allows for use in new applicability domains. For example it is possible to try different hypothesis regarding chemical structures with instant feedback, which for the first time makes site-of-metabolism prediction a feasible methodology to integrate with a chemical drawing application. The combination of MetaPrint2D and Bioclipse greatly simplifies site-of-metabolism predictions in the eyes of scientists. The MetaPrint2D algorithm has proven to be extremely fast compared to other available tools, and opens up the possibility to perform site-of-metabolism predictions on a large scale. The integration into Bioclipse extends the usability and accessibility of MetaPrint2D, and in contrast to existing tools, predictions can now be done on regular laptops at execution times that are considerable less than one second per molecule. The implementation does not require access to remote computers, and the accuracy of the predictions to the predecessor of MetaPrint2D has been shown to be on par with other tools in the field [4].

The intuitive user interface in Bioclipse enables MetaPrint2D to be used by a variety of people in the drug discovery process, ranging from computational chemists to experts in drug metabolism. Molecules can easily be drawn in the chemical editor of Bioclipse and then subjected to MetaPrint2D prediction, or predictions can be made on all molecules in a file. The real time execution provided in single molecule mode allows for the user to try different hypothesis with very little effort, and get instant feedback of the predicted site of metabolism for a change in the chemical structure of a compound. The option to visualise multiple predictions in a tabular view allows for simultaneously working with collections of compounds with the same functionality, and easy switching to editing individual compounds. For larger collections or when visual inspection is not desired, the batch functionality for running predictions on a file with results stored as properties is an appealing option. For more advanced users, Bioclipse provides the means to call MetaPrint2D from within a Bioclipse script and integrate it with other applications. The advanced provisioning system of Bioclipse greatly facilitates updates to the implementation and the databases of the MetaPrint2D plugin.

Open-source software provides excellent opportunities to extend and combine different software projects. The application presented here is an example of this and there are several benefits of this: 1) It is completely free of charge, 2) Anyone who has ideas on how to improve the algorithms can either contribute to any of the two projects or take all available software and tailor it for their own specific needs, and 3) The access to the source code ensures reliability and builds trust. There are no hidden details and everything can be reproduced. Both MetaPrint2D and Bioclipse are released under open source licenses and are completely free of charge.

## Competing interests

The authors declare that they have no competing interests.

## Authors' contributions

LC came up with the original idea to include MetaPrint2D within Bioclipse as a plugin. He also drafted the manuscript and developed the MetaPrint2D algorithm. OS designed and implemented the MetaPrint2D plugin and drafted the manuscript. SA drafted the manuscript and developed and implemented MetaPrint2D. RCG and SB developed MetaPrint2D. All authors read and approved the final manuscript.

## Appendix A: Availability

### Getting Bioclipse and MetaPrint2D

A Bioclipse plugin enabling the use of the MetaPrint2D library from within Bioclipse is available. Bioclipse must be downloaded and installed from http://www.bioclipse.net. The easiest way to install MetaPrint2D is to use the option Software Updates from the Help menu in Bioclipse. Bioclipse will then contact the update server and present the user with a list of features that is available for installation. By checking MetaPrint2D and clicking "Add required plugins", the MetaPrint2D functionality is available after restart (a new menu, a toolbar and context menu actions) when browsing/editing chemical structures and files.

The source code of the MetaPrint2D library is available from http://sourceforge.net/projects/metaprint2d/.

## License

Bioclipse is released under Eclipse Public License (EPL) [15]. EPL is a flexible open source license that ensures core plugins will remain open source, but sets no constraints on external plugin licensing. More information can be found on the Bioclipse home page. MetaPrint2D 1.0 is released under GNU Lesser General Public License (LGPL) [16].
